# MRI arterial spin labeling in evaluating hemorrhagic transformation following endovascular recanalization of subacute ischemic stroke

**DOI:** 10.3389/fnins.2023.1105816

**Published:** 2023-03-03

**Authors:** Liheng Wu, Yanghui Liu, Liangfu Zhu, Tianxiao Li, Li’na Wang, Yang Zhang, Zhilong Zhou, Ying Xing, Meiyun Wang, Bulang Gao

**Affiliations:** ^1^Department of Cerebrovascular Diseases, National Advanced Stroke Center, Henan Provincial People’s Hospital, People’s Hospital of Henan University, Zhengzhou, China; ^2^Department of Imaging, Henan Provincial People’s Hospital, People’s Hospital of Henan University, Zhengzhou, China

**Keywords:** ischemic stroke, arterial spin labeling, blood-brain barrier permeability, hemorrhagic transformation, arterial recanalization

## Abstract

**Objective:**

To investigate the value of the MRI arterial spin labeling (ASL) in evaluating the blood-brain barrier permeability of anterior circulation ischemic lesions in subacute ischemic stroke (SIS) and the risk of hemorrhage transformation (HT) after endovascular recanalization.

**Materials and methods:**

Patients with anterior circulation SIS treated with endovascular recanalization were prospectively enrolled. The imaging presentations in the MRI ASL sequences, dynamic contrast-enhanced (DCE) sequence, and Xper CT were studied. The relative cerebral blood flow (rCBF), volume transfer constant (Ktrans), and the weighted Kappa coefficient (rKtrans) were analyzed.

**Results:**

Among 27 eligible patients, HT occurred in 7 patients (25.92%). Patients with HT had significantly higher rCBF value (1.56 ± 0.16 vs. 1.16 ± 0.16), Ktrans, (0.08 ± 0.03 min vs. 0.03 ± 0.01 min) and rKtrans (3.02 ± 0.89 vs. 1.89 ± 0.56). The ASL imaging sequence had a high consistency with the DCE sequence and Xper CT with a high weighted Kappa coefficient of 0.91 for the DCE sequence and 0.70 for the Xper CT imaging. The DCE sequence was also highly consistent with the Xper CT in imaging classification with a high weighted Kappa coefficient of 0.78. The rCBF value in the 21 patients with the subcortical and basal ganglia infarction was significantly lower than that in the other 6 patients with the cortical infarction (1.222 ± 0.221 vs. 1.413 ± 0.259, *t* = 1.795, *P* = 0.004).

**Conclusion:**

The MRI ASL sequence has an important role in evaluating the blood-brain barrier permeability and the risk of hemorrhagic transformation of anterior circulation SIS following endovascular recanalization.

## Introduction

For patients with subacute ischemic stroke caused by symptomatic intracranial atherosclerotic stenosis or occlusion, endovascular recanalization can be safely performed with good effects after strict selection of appropriate indications, especially within two weeks after symptom onset (Topakian et al., 2007). Nonetheless, some patients may still have hemorrhagic transformation after endovascular recanalization (Arba et al., 2021). Hemorrhagic transformation is one of the main complications after endovascular treatment of ischemic stroke and may seriously affect the prognosis of these patients. How to effectively predict the hemorrhagic transformation is of great significance for guiding the endovascular treatment of ischemic stroke.

Destruction of the blood-brain barrier is one of the major pathological mechanisms of hemorrhagic transformation after ischemic stroke. To some extent, detection of the blood-brain barrier permeability can predict occurrence of hemorrhage transformation after endovascular recanalization of occluded arteries. However, because the damage and dynamic repair of the blood-brain barrier are highly individualized, how to accurately detect the permeability of the blood-brain barrier becomes the key. It has been reported that dynamic contrast-enhanced (DCE) sequence magnetic resonance imaging (MRI) can be applied to quantitatively assess the permeability of the blood-brain barrier according to its sequence parameter, the volume transfer constant (Ktrans) across the blood-brain barrier [3–4]. The Ktrans is proportional to the barrier permeability, but evaluation of the blood-brain barrier with the Ktrans requires injection of contrast agents, which may lead to some adverse reactions (Raja et al., 2018; Varatharaj et al., 2019). Arterial spin labeling (ASL) MRI examination only marks the patient’s own water proton imaging without contrast agent injection. In the study of acute ischemic stroke, it was found that local measurement of the ASL’s sequence parameters relative to the cerebral blood flow (rCBF) could also be used to evaluate the permeability of the blood-brain barrier, and the rCBF value > 1.3 could effectively predict the presence of hemorrhagic transformation after endovascular treatment (Nogueira et al., 2015; Niibo et al., 2017). It was hypothesized that ASL could be used to efficiently evaluate the permeability of blood-brain barrier in patients with cerebral infarction and predict hemorrhagic transformation after endovascular recanalization of stenotic or occluded arteries. This study was thus performed to investigate the value of ASL in assessing the permeability of blood-brain barrier and predicting hemorrhagic transformation after the endovascular treatment in patients with subacute anterior circulation ischemic stroke caused by severe intracranial atherosclerotic stenosis or occlusion. This may provide more technical support for evaluation of the brain tissue status during the perioperative period, facilitate targeted classification and staging treatment of patients, and prevent hemorrhagic transformation.

## Materials and methods

### Subjects

This prospective one-center study was carried out after approval by the ethics committee of our hospital (2020108), and all patients or their family members had signed the informed consent to participate. Between January 2021 and September 2021, consecutive patients with subacute ischemic stroke of the anterior circulation who received endovascular recanalization were prospectively enrolled. The inclusion criteria were age ≥ 18 years, the modified Rankin scale (mRS) score before stroke onset < 3, anterior circulation ischemic stroke confirmed by clinical and imaging examinations and managed with medication for 2–3 weeks after stroke onset, responsible arteries being the middle cerebral artery and the intra- or extracranial segments of the internal carotid artery with the stenosis ≥ 70% or complete occlusion, examination of the MRI ASL sequence and DCE sequence 24 h before and immediately after endovascular balloon angioplasty alone or stent angioplasty, and Xper CT being performed immediately after the endovascular treatment. The exclusion criteria were non-intracranial atherosclerotic stenosis caused by Moyamoya disease, Moyamoya syndrome or vasculitis, a previous ischemic lesion ≥ 1.5 cm in diameter in the responsible vascular basin of this ischemic stroke lesion, presence of hemorrhagic transformation before endovascular treatment confirmed by imaging examination, suffering from malignant tumors, and complicated with renal insufficiency or allergy to gadolinium contrast agent.

### Imaging examination

All the MRI ASL sequence, DCE sequence and Xper CT examinations were completed on the “one-stop multimodal image fusion stroke treatment platform.” The platform was composed of a 3.0T MR scanner (MAGNETOM Skyra), a 64 row multi-slice CT scanner (SOMATOM Definition AS), and a dual C-arm DSA system (Artis Q biplane) (Siemens, Germany). In MRI, images were collected with a 8-channel head coil. The repeatition time of axial T1WI fast echo sequence was 4.1 ms, an echo time 120 ms, and a visual field 24 cm × 24 cm. The DWI sequence had a repetition time 4200 ms, an echo time 100 ms, a b value 1000 s/mm^2^, and a field of view 24 cm × 24 cm. The repeat time of 3D-ASL sequence was 4564 ms, an echo time 10.5 ms, a matrix 128 × 128, a field of view 24 cm × 24 cm, layer thickness 3 mm, excitation times 2 times, delay time after labeling 1550 ms, 2550 ms (multi-delay technique), and scanning time 447 s. The repetition time of DCE sequence was 5.3 ms, an echo time 1.9 ms, layer thickness 5 mm, no spacing, a matrix 256 × 256, a field of view 24 cm × 24 cm, a reverse angle 15°, time resolution 380/35 s, tracer kinetic model of the Tofts model, and same scanning and positioning parameters as the above sequence. The number of layers was 40, including 35 times of acquisition. After the third image acquisition, the contrast agent (Gdap injection, 20 mL/tube, Guangzhou Kangchen Pharmaceutical Co., Ltd.) was injected through the elbow vein at a rate 4 mL/s and a dose 0.1 mmol/kg, and the scanning time was 380 s with a temporal resolution of 380/35 s.

### Imaging post-processing

The imSTROKE software (Nanjing Yuexi Medical Technology Co., Ltd., Nanjing, China) was used for the ASL image *post*-processing. Firstly, ASL, DWI and apparent dispersion coefficient (ADC) data were input before rigid registration of the ASL data with the DWI and ADC data. After the DWI and ADC sequences were classified with the artificial intelligence, the infarct core region was automatically calculated, and the final region of interest (ROI) was obtained through morphological processing. The contralateral healthy region corresponding to the infarcted ROI was selected to obtain the median value of the pixel in the region as the reference value before calculating the rCBF and converting it into pseudo-color display in the ROI. The Siemens Syngo workstation software was used to select the corresponding ROI on the DCE sequence according to the ROI on the ASL sequence before automatically measuring and recording the Ktrans value and rKtrans value (the ratio of the ROI Ktrans value on the infarcted side to that on the healthy side). The rCBF parameters in the MRI ASL sequence and the Ktrans parameters in the DCE sequence were used, respectively, to reflect the permeability of blood-brain barrier and local hyperperfusion state. The Ktrans is a parameter of the MRI DCE sequence and calculated as the mean value in a certain ROI region, and it is a volume transfer constant across the blood-brain barrier (Raja et al., 2018; Varatharaj et al., 2019) and can be applied to quantitatively assess the permeability of the blood-brain barrier because it is proportional to the barrier permeability (Raja et al., 2018; Varatharaj et al., 2019).

### Imaging classification of hemorrhagic transformation

**FIGURE 1:**
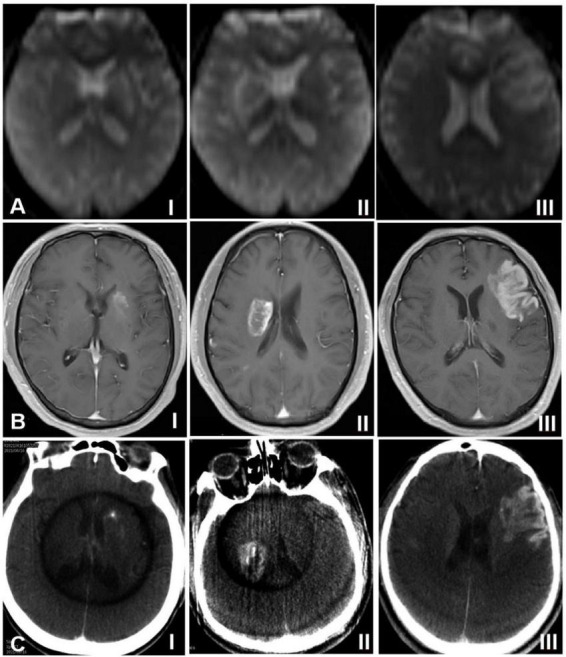
Classification of hemorrhagic transformation on arterial spin labeling imaging (ASL), dynamic contrast-enhanced (DCE) sequence of magnetic resonance imaging, and Xper CT. **(A)** ASL sequence imaging, **(B)** DCE sequence imaging, and **(C)** Xpert CT imaging. I indicates type I, II indicates type II, and III indicates type III.

On the ASL and DCE sequences, hemorrhagic transformation was presented as areas of high signals (high signals on the ASL sequence represented an increase of local rCBF, and high signals on the DCE sequence represented an increase of contrast agent exudation, which reflected the increase of blood-brain barrier permeability) (Nogueira et al., 2015; Niibo et al., 2017; Raja et al., 2018; Varatharaj et al., 2019). According to the high-signal distribution range of hemorrhagic transformation, the imaging presentations of hemorrhagic transformation were classified as follows: Type I: the high signal areas were distributed in dots or stripes around the edge of the cerebral infarction focus; Type II: The high signal areas in the cerebral infarction focus were distributed in patches, with the volume less than 30% of the cerebral infarction focus; Type III: The high signal areas in the cerebral infarction focus were distributed in sheets, with the volume ≥ 30% of the cerebral infarction focus (Figure 1). On Xper CT imaging, high-density lesions in low-density background infarction area immediately after endovascular treatment were hemorrhagic transformation. According to the range of high-density lesions shown on the Xper CT imaging immediately after endovascular treatment (Sandoval and Witt, 2008), the imaging classification was as follows: Type I: the contrast agent exuded around the edge of the cerebral infarction, and high-density lesions were distributed in spots or strips; Type II: the contrast agent exuded in the cerebral infarction focus, and high-density lesions were distributed in patches, with the volume less than 30% of the cerebral infarction focus; Type III: the contrast agent exuded in the cerebral infarction focus, and high-density lesions were distributed in sheets, with the volume ≥ 30% of the cerebral infarction focus (Figure 1). The ASL, DCE and Xper CT imaging classifications were independently assessed by two imaging experts with senior professional titles who were not aware of the diagnosis, treatment and prognosis of the disease. If disagreements arose, consultation to a third senior physician was performed to reach a consensus.

In addition, all patients were reexamined with plain CT scan 24–48 h after endovascular treatment. Presence of high-density lesions on the low-density background layer of cerebral infarction was defined as the occurrence of hemorrhagic transformation, and the Heidelberg classification standard was used for the classification of three entities (von Kummer et al., 2015): HI type 1 (scattered small petechiae with no mass effect), HI type 2 (confluent petechiae with no mass effect), and PH 1 Hematoma within the infarcted tissue occupying < 30% with no substantive mass effect.

### Treatment plan

Before endovascular recanalization, all patients were treated with dual antiplatelet therapy. After successful arterial puncture during the endovascular procedure, intravenous bolus loading of unfractionated heparin (70 IU/kg) was administered. During the procedure, 1000 IU of unfractionated heparin was added every hour. Glycoprotein II b/III α receptor antagonists such as tirofiban were not used during the perioperative period.

### Statistical analysis

The SPSS 25.0 software (IBM, Chicago, IL, USA) was used for data analysis. Measurement data were expressed as the mean ± standard deviation if in the normal distribution and tested with the *t*-test but as the median (interquartile range) if in the skew distribution and tested with the Mann–Whitney U test. Categorical variables were presented in frequency and percentages and tested with the Chi square test or Fisher’s exact probability test. The weighted Kappa coefficients were used to check the consistency among ASL, DCE and Xper CT imaging classifications. The difference was statistically significant if *P* < 0.05.

## Results

### General information

**FIGURE 2:**
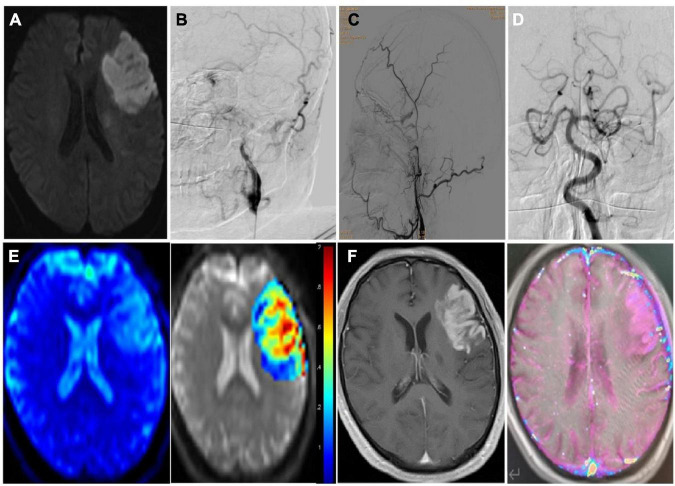
Imaging data of a patient with subacute ischemic stroke with hemorrhagic transformation after endovascular therapy. **(A)** Seven days after stroke onset, diffusion weighted imaging sequence of MRI shows subacute cerebral infarction in left frontal temporal lobe. **(B–D)** Cerebral angiography revealed long-segment occlusion of the left internal carotid artery, but the left middle cerebral artery and its branches remain patent. **(E)** The pseudo color image (left) and *post*-processing image (right) of the ASL (arterial spin labeling) sequence are shown with the cerebral blood flow of 1.9. **(F)** The original image (left) and the *post*-processing image (right) of the DCE (dynamic contrast enhanced) MRI sequence are shown with the Ktrans of 0.16/min).

**FIGURE 3:**
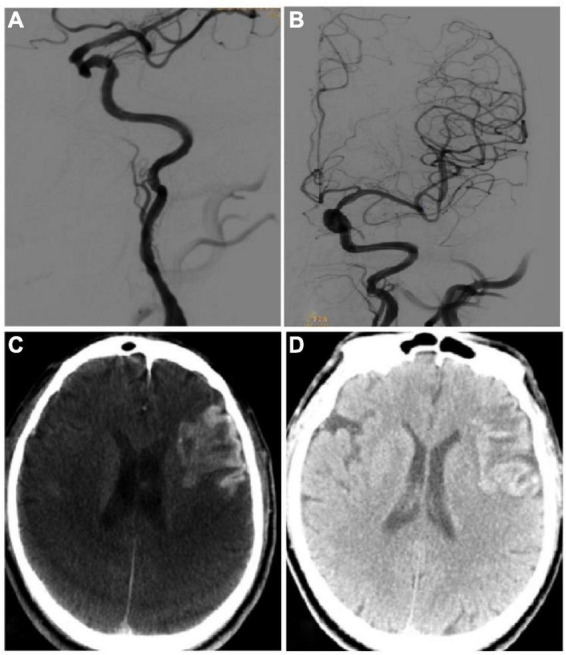
Imaging data of the same patient as in Figure 2. **(A,B)** On the 20th day after symptom onset, endovascular recanalization was performed to recanalize the long-segment occlusion of the left internal carotid artery, and two resolute stents (4.0 mm × 33 mm and 4.0 mm × 36 mm) were deployed in sequence. After recanalization, the occluded internal carotid artery was successfully recanalized. **(C)** Xper CT immediately after the surgery showed that the imaging classification of hemorrhagic transformation was type III. **(D)** Hemorrhage transformation was detected by CT plain scan on the next day after surgery.

Twenty-seven patients were enrolled, including 17 males and 10 females, with a mean age of 62.37 ± 7.76 (61–76) years (Table 1). Hypertension was present in 19 cases, diabetes mellitus in 9, hyperlipidemia in 21, and stroke history in 14. The responsible vessels were the middle cerebral artery in 13 cases, the internal carotid artery intracranial segment in 7 cases, and the extracranial segment of the internal carotid artery in 7 cases. The cerebral infarction site was in the cortex in 6 cases and in the subcortical and basal ganglia in 21 cases (Figures 2, 3). Severe arterial stenosis was presented in 14 cases, and occlusion in 13 cases. In endovascular treatment, balloon angioplasty alone was performed in 4 cases, and stent angioplasty in 23 cases.

**TABLE 1:** Clinical and imaging data in the HT and non-HT groups.

Variables		Total	HT	Non-HT	Z/t/χ 2	*P*
Gender (M/F)		17/10	6/1	11/9	–	0.204
Age [M (Q1, Q3)]		63.5 (63, 67)	63 (50.25, 65)	64 (63, 67.50)	−1.518	0.129
Risk factors (n)	Hypertension	19	4	15	–	0.633
	Diabetes mellitus	9	3	6	–	0.653
	Hyperlipidemia	21	6	15	–	1
	History of stroke	14	4	10	–	1
Responsible arteries (n)	MCA	13	4	9	0.678	0.712
	Intracranial ICA	7	6	1		
	Extracranial ICA	7	5	2		
Cerebral infarction (n)	Cortex	6	2	2	–	0.633
	Subcortical and basal ganglia	21	5	16		
Degree of vascular disease (n)	Severe stenosis	14	2	9	–	0.678
	Occlusion	13	3	8		
Endovascular treatment (n)	Balloon angioplasty	4	0	3	–	1
	Stent angioplasty	23	5	14		
Time from onset to imaging (d)		18.96 ± 1.81 (14–21)	18.29 ± 1.25 (16–20)	19.2 ± 1.94 (14–21)	−2.410	0.26
rCBF (x ± s)		1.26 ± 0.234 (0.8–1.9)	1.56 ± 0.16 (1.4–1.9)	1.16 ± 0.16 (0.8–1.42)	5.943	<0.001
rKtrans (x ± s)		2.18 ± 0.82 (1–4.3)	3.02 ± 0.89 (2.01–4.3)	1.89 ± 0.56 (1–2.86)	3.371	<0.001
Ktrans [/min, M(Q1, Q3)]		0.04 ± 0.02 (0.01–0.16)	0.08 ± 0.03 (0.04–0.16)	0.03 ± 0.01 (0.01–0.06)	5.291	<0.001
ASL (n)	Type I	8	0	8	15.20	0.26
	Type II	11	1	10		
	Type III	8	6	2		
DCE (n)	Type I	9	0	9	12.10	0.21
	Type II	11	2	9		
	Type III	7	5	2		
Xper CT (n)	Type I	12	0	12	13.6	0.24
	Type II	11	4	7		
	Type III	4	3	1		

HT, hemorrhagic transformation; MCA, middle cerebral artery; ICA, internal carotid artery; ASL, arterial spin labeling; DCE, dynamic contrast-enhanced magnetic resonance sequence; rCBF, relative cerebral blood flow; Ktrans, volume transfer constant; rKtrans, relative volume transfer constant.

The time from onset to imaging examination was 18.96 ± 1.81 (14–21) days. On the ASL imaging, type I was in 8 cases, type II in 11, and type III in 8, with an average rCBF of 1.32 (1.120, 1.510). On the DCE imaging, type I was in 9 cases, type II in 11, and type III in 7. The Ktrans was 0.04 ± 0.02 (0.01–0.16), and the rKtrans was 2.18 ± 0.82 (1–4.3). On the Xper CT imaging, type I was in 12 cases, type II in 11, and type III in 4.

### Clinical and imaging presentations

Among 27 patients, 7 (25.92%) patients had hemorrhagic transformation after endovascular recanalization, all of whom were of Heidelberg type II (Figures 2, 3 and Table 1). There were no significant (*P* > 0.05) differences in the gender, age, high risk factors, location of cerebral infarction, distribution of responsible vessels, extent of disease, modes of endovascular treatment, time from onset to imaging examination, and imaging types on ASL sequence, XperCT and DCE MRI between patients with and those without hemorrhagic transformation. However, the rCBF value (1.56 ± 0.16 vs. 1.16 ± 0.16), Ktrans, (0.08 ± 0.03 vs. 0.03 ± 0.01) and rKtrans (3.02 ± 0.89 vs. 1.89 ± 0.56) were all significantly (*P* < 0.05) higher in patients with than those without hemorrhagic transformation.

### Consistency between the ASL, DCE, and Xper CT imaging classifications

The imaging classification was consistent on the ASL sequence with that on the DCE sequence in 25 patients, but inconsistent in only 2 patients, with a weighted Kappa coefficient of 0.91 (95% CI: 0.79–1.03, *P* < 0.001), indicating strong consistency (Table 2). The imaging classification was consistent on the ASL sequence with that on the Xper CT imaging in 19 patients, but not in the other 8 patients, resulting in a weighted Kappa coefficient of 0.70 (95% CI: 0.50–0. 89, *P* < 0.001), with strong consistency (Table 2). The imaging classification was consistent on the DCE sequence with that on the Xper CT imaging in 19 patients, but not in the other 8 patients, leading to a weighted Kappa coefficient of 0.78 (95% CI: 0.60–0.96, *P* < 0.001), with strong consistency (Table 3).

**TABLE 2:** Consistency of imaging types between ASL and DCE MRI.

ASL imaging	DCE imaging				Xper CT imaging			
	**Type I**	**Type II**	**Type III**	**Total**	**Type I**	**Type II**	**Type III**	**Total**
Type I	8	0	0	8	8	0	0	8
Type II	1	11	0	11	4	7	0	11
Type III	0	0	7	8	0	4	4	8
Total	9	11	7	27	12	11	4	27

ASL, arterial spin labeling; MRI, magnetic resonance imaging; DCE, dynamic contract enhanced; CT, computed tomography.

**TABLE 3:** Consistency of imaging types between Xper CT and DCE MRI.

DCE imaging	Xper CT imaging			Total
	**Type I**	**Type II**	**Type III**	
Type I	8	1	0	9
Type II	4	7	0	11
Type III	0	3	4	7
Total	12	11	4	27

CT, computed tomography; MRI, magnetic resonance imaging; DCE, dynamic contract enhanced.

### rCBF and Ktrans values in different cerebral infarction sites

The rCBF value in the 21 patients with the subcortical and basal ganglia infarction was significantly lower than that in the other 6 patients with the cortical infarction (1.222 ± 0.221 vs. 1.413 ± 0.259, *t* = 1.795, *P* = 0.004), however, no significant difference was found in the Ktrans (0.04 ± 0.02 vs. 0.06 ± 0.03, *P* = 0.13) or rKtrans (2.08 ± 0.79 vs. 2.54 ± 0.89, *P* = 0.23) between the two groups.

## Discussion

In this study investigating the value of the ASL imaging sequence in evaluating the blood-brain barrier permeability of anterior circulation ischemic lesions in subacute ischemic stroke and in assessing the risk of hemorrhage transformation after endovascular recanalization, it was found that the ASL sequence had a strong role in evaluating the blood-brain barrier permeability in patients with anterior circulation subacute ischemic stroke and that Type III ASL imaging presentation may have a high prediction effect on the risk of hemorrhagic transformation.

For patients with subacute ischemic stroke caused by severe stenosis or occlusion of large intracranial vessels, endovascular recanalization at an appropriate time can benefit most patients and effectively reduce the poor prognosis. However, some complications after endovascular arterial recanalization cannot be ignored. Among them, hemorrhage transformation is one of the serious and even fatal complications after endovascular treatment of ischemic stroke, and its occurrence is closely related to the increase of blood-brain barrier permeability (Sandoval and Witt, 2008). Damage of the blood-brain barrier will cause large-molecular neurotoxic proteins in the blood to leak out into the brain tissue gap, mediating inflammatory reaction to cause perivascular interstitial edema, neuronal and nerve fiber damage and subsequent blood leakage out of the cerebral arteries (or bleeding), which is the hemorrhagic transformation from ischemic stroke after endovascular treatment. This hemorrhagic transformation cannot be directly measured, but can be indirectly reflected by measurement of the blood-brain barrier permeability. In addition, increased permeability in the blood-brain barrier caused by cerebral ischemic injury appears not only in the acute phase, but also in the subacute phase and the early stage of angiogenesis associated with the recovery of ischemic stroke (Jiang et al., 2012). Therefore, quantitative analysis of the blood-brain barrier permeability is a promising approach to predict the risk of hemorrhagic transformation in patients with subacute ischemic stroke.

The blood-brain barrier is crucial for maintaining a stable cellular environment in the central nervous system for regulation of cellular and solute traffic between the blood and the brain. Permeability of the blood-brain barrier is a physiological phenomenenon in the healthy state but increases with aging and in the disease status (Elwood et al., 2017). The first blood-brain interface is formed by the endothelial cells that have tight junction proteins, and the next layer is formed by the basal lamina which is composed of extracellular matrix proteins and surrounded by the astrocytic foot processes with embedded pericytes. Under normal conditions, the interface between brain tissues and blood is a relatively limited “barrier” to non-lipid molecules and has low permeability. Besides the tight junction proteins, there are also transport or carrying molecules to facilitate transport into the brain, and enzymes may exist to degrade substances and prevent transport into the brain (Raja et al., 2018). When the blood-brain barrier is damaged in brain tumors, stroke or multiple sclerosis, large proteins may leak into the brain tissue and cerebrospinal fluid, which can be detected with CT or MRI. Disruption of the blood-brain barrier in chronic vascular disease is associated with hypoxia-induced inflammation (Raja et al., 2018). One potential condition to involve chronic vascular changes is secondary to long-standing elevated blood pressure, which may lead to stenosis of the vascular lumen, limiting blood flow and producing hypoxia. This finding has been proved by animal studies of hypertensive rats (Weaver et al., 2014) and human studies (Fernando et al., 2006) with hypoxia-induced factors being found in the brains of patients with vascular dementia. Nonetheless, measurement of the blood-brain barrier permeability is not straightforward. The ratio between the cerebrospinal fluid and the serum albumin is a well-established approach to evaluate the barrier permeability, but is invasive and may not reliably reflect the real permeability, with a great influence from the cerebrospinal fluid flow (Varatharaj et al., 2019). Neuro-imaging with use of an intravenous injection of tracer is an approach to measure the blood-brain barrier permeability, and positron emission tomography has also been used in this aspect (Varatharaj et al., 2019). Nonetheless, some disadvantages in these approaches have precluded the widespread application, including the suboptimal resolution and radioactivity.

Currently, a variety of neuroimaging techniques can be used to obtain the volume of cerebral infarction, the perfusion status of infarction focus, the degree of vascular disease, and the permeability of diseased vascular wall to predict the risk of hemorrhagic transformation in patients with ischemic stroke. The use of DCE sequence MRI is a commonly used imaging method to detect the permeability of the blood-brain barrier before endovascular treatment. Its parameter Ktrans represents the speed of contrast agent penetrating from the cerebral vessels into the interstitium and is calculated by measuring the mean Ktrans in a certain ROI region. It can reflect the concentration and flow of contrast agent in the blood of cerebral tissue. Thus, it can be used as an indicator of the blood-brain barrier permeability, and has been shown to be able to sensitively detect the changes of the blood-brain barrier permeability at the early stage of cerebral ischemia (2 to 3 h) (Jiang et al., 2005). However, it is undeniable that the sensitivity of MRI based on the gadolinium chelate contrast agent for ischemic stroke is relatively low, and adverse reactions such as nephrogenic systemic fibrosis and intracranial gadolinium deposition may occur. Immediate plain CT scanning after endovascular treatment can also detect contrast agent extravasation in the local cerebral infarction, so as to judge the permeability of the diseased vascular wall, extent of blood-brain barrier damage and risk of hemorrhagic transformation. Nonetheless, these findings are after rather than before endovascular recanalization, which is not conducive to early intervention. In addition, although the exudation of contrast agent on Xper CT after surgery had been demonstrated to be associated with the risk of hemorrhagic transformation (Seevinck et al., 2010; Chen et al., 2020), only qualitative analysis had been performed without classifying the exudation characteristics or quantity.

ASL is the only non-invasive technique using endogenous tracers in perfusion imaging. In a study investigating the MRI ASL sequence imaging in 27 patients with acute ischemic stroke after endovascular treatment (Shimonaga et al., 2019), it was found that among 13 patients with local hyperperfusion areas, 8 patients had hemorrhagic transformation after endovascular recanalization, and the prognosis of these patients was poor, suggesting that the increase of local cerebral blood flow in cerebral infarction can be used to predict the occurrence of hemorrhagic transformation. In our study, the image scanning of the ASL sequence, DCE sequence and Xper CT was completed on the “one-stop multimodal image fusion and stroke treatment platform” dedicated to stroke diagnosis and treatment, which not only helped to achieve unification of relevant parameters, but also reduced the time interval of image inspection, ensuring the accuracy and repeatability of image data to the maximal extent. In our study, the DCE sequence and Xper CT were used to cross verify the evaluation effect of the ASL sequence on the blood-brain barrier permeability of cerebral infarction focus in patients with subacute ischemic stroke and the predictive value of hemorrhage transformation. The results showed that in the same ROI, the rCBF and Ktrans values in patients with hemorrhagic transformation were significantly higher than those without hemorrhagic transformation.

After further classification of the high signal distribution on the ASL and DCE sequence and the high-density image characteristics on the Xper CT, it was found that the ASL imaging sequence had a high consistency with the DCE imaging sequence and the Xper CT with a Kappa coefficient of 0.91 and 0.70, respectively. Consequently, the ASL sequence may be just like the DCE sequence and the Xper CT, playing a strong role in assessing the blood-brain barrier permeability in the cerebral infarction area of subacute ischemic stroke. The risk of hemorrhagic transformation in patients with ASL type III was relatively high. The synergy of the three imaging tools may be able to make a more accurate prediction of postoperative hemorrhagic transformation, thus providing more objective evidence for guiding the classification, staging and individualized treatment of stroke patients.

The location of contrast agent extravasation is more valuable than the extravasation itself in predicting the hemorrhagic transformation. Because contrast agent is mostly injected near the opening of the lenticular artery in the middle cerebral artery trunk, contrast agent extravasation limited to the basal ganglia is common, but its value in predicting the hemorrhagic transformation is low. Extravasation of contrast agent in the cortex may indicate extensive damage of the blood-brain barrier, serious reperfusion injury, and increased risk of symptomatic hemorrhagic transformation (Kim et al., 2015). However, further analysis in our study found no significant difference in the Ktrans value between patients with subcortical and basal ganglia infarction and those with cortical infarction (*P* > 0.05), even though the rCBF value of the former was significantly lower than that of the latter. This may indicate differences in the blood-brain barrier permeability between infarcted tissues in different parts of subacute ischemic stroke, which may be associated with different ischemic tolerance of the blood-brain barrier in different parts of brain tissue with subacute ischemic stroke as well as different repair efficiency of the blood-brain barrier permeability. It has been found that the ischemic focus of subacute ischemic stroke will have functional neurovascular remodeling, which is accompanied by neovascularization, and establishment of collateral circulation will accordingly increase blood perfusion in the area with neovascularization (Bokkers et al., 2009; Yang and Torbey, 2020). In patients with subacute ischemic stroke, the leptomeningeal collateral branch is easier to be established in patients with cortical infarction than in patients with subcortical and basal ganglia infarction, which may lead to the earlier and more perfect repair of the blood-brain barrier in patients with cortical infarction.

Our study also suggested that the ASL and DCE sequences have different abilities in detecting the blood-brain barrier permeability in different parts of subacute ischemic stroke, which may be related to the difference in imaging characteristics between the two sequences. The ASL sequence is marked by water protons which are more sensitive to changes in the blood-brain barrier permeability than gadolinium ions (Lin et al., 2018; Mahroo et al., 2021). Therefore, the ASL sequence is more sensitive to changes in the blood-brain barrier permeability than the DCE sequence. Moreover, in our study, the ASL sequence imaging used the “multiple delay labeling” technology, which is easy to capture long delayed signals in different parts of tissues (Mato Abad et al., 2016), resulting in more sensitivity of the ASL sequence to changes in the blood-brain barrier permeability in different parts than that of the DCE sequence. Based on the results of our study and the above analysis, the ASL sequence imaging using the “multiple delay labeling” technology in clinical work may be more suitable to detecting the nervous system diseases with slight changes in the blood-brain barrier permeability.

Some limitations existed in our study, including enrollment of Chinese patients only, one-center study design and a small cohort of patients due to the strict inclusion and exclusion criteria, which may all affect the bias of the outcome. Further prospective, randomized, controlled clinical trials with multicenters involved and a large sample size are needed to verify the outcomes of this study.

To sum up, application of the ASL sequence and its advanced *post*-processing technology can effectively assess the blood-brain barrier permeability in patients with subacute ischemic stroke in the anterior circulation and predict the risk of hemorrhagic transformation following endovascular recanalization, thus conducive to selection of surgical timing for different patients, reduction of perioperative complications, and achievement of real staging and individualized treatment for the patients.

## Data availability statement

The original contributions presented in this study are included in this article/supplementary material, further inquiries can be directed to the corresponding author.

## Ethics statement

The studies involving human participants were reviewed and approved by the Ethics Committee of Henan Provincial People’s Hospital. The patients/participants provided their written informed consent to participate in this study.

## Author contributions

LZ and BG: study design. LHW, YL, LNW, YZ, ZZ, YX, and MW: data collection. LHW, LNW, YL, LZ, and TL: data analysis. YL: writing of the original version. BG: revision. TL: supervision. All authors contributed to the validation article and approved the submitted version.
